# Coordination of phase precession through feed-forward topologies in the hippocampus

**DOI:** 10.1186/1471-2202-14-S1-P206

**Published:** 2013-07-08

**Authors:** Jorge Jaramillo, Robert Schmidt, Richard Kempter

**Affiliations:** 1 Institute for Theoretical Biology, Department of Biology, Humboldt-Universität zu Berlin, 10115 Berlin, Germany; 2 Bernstein Center for Computational Neuroscience Berlin, 10115 Berlin, Germany; 3 Neurowissenschaftliches Forschungszentrum, Charité-Universitätsmedizin Berlin, 10117 Berlin, Germany; 4 Department of Psychology, University of Michigan, Ann Arbor, MI 48104, USA

## 

The process of faithfully retrieving episodes from our memory requires a neural mechanism capable of initially forming ordered and reliable behavioral sequences. These behavioral sequences take place on a timescale of seconds or more whereas the timescale of neural plasticity and learning is in the order of tens of milliseconds. To shed light on this dilemma, we turn to studies of hippocampal place cells in rodents, i.e., cells that selectively increase their firing rates in locations of the environment known as the place fields. Within a field, the firing phases of a place cell precess monotonically relative to the ongoing theta rhythm. This phenomenon, termed "phase precession" [1], leads to a temporally compressed representation of the behavioral sequences experienced by the rodent [2], and the compressed timescale matches the requirements of neural plasticity.

Phase precession has been observed in several regions of the hippocampus and entorhinal cortex, but it remains a mystery whether phase precession emerges independently in each region, or conversely, whether phase precession can be "inherited" from an upstream neuronal population. Here, we show how the simple feed-forward topologies in the hippocampal formation assist in propagating phase precession to different regions of the hippocampus and adjacent structures. We explain how the place-selective responses of a given cell are influenced by both excitation and inhibition and show that these responses are consistent with intracellular [3] and extracellular recordings in-vivo during phase precession [4][5]. Our results suggest that the presence of phase precession in different stages of the hippocampal circuit is indicative of a common source, a fact that can help us better understand sequence coding within the hippocampus.
